# Evaluation of Stress Response under a Standard Euthanasia Protocol in Horses Using Analysis of Heart Rate Variability

**DOI:** 10.3390/ani10030485

**Published:** 2020-03-13

**Authors:** Heidrun Gehlen, Johanna Loschelder, Roswitha Merle, Maike Walther

**Affiliations:** 1 Equine Clinic, Freie Universitaet Berlin, 14195 Berlin, Germany; Johanna.Loschelder@fu-berlin.de (J.L.); maike@waltherbochum.de (M.W.); 2 Institute of Veterinary Epidemiology, Veterinary Department, Freie Universitaet Berlin, 14195 Berlin, Germany; roswitha.merle@fu-berlin.de

**Keywords:** equine, euthanasia, heart rate variability, stress

## Abstract

**Simple Summary:**

In our study, we examined different stress factors during euthanasia in horses. Therefore, we measured the heart rate variability (HRV), which is a method to determine stress in various species. The HRV was determined in 40 horses undergoing euthanasia due to various reasons, at different times and locations throughout the clinic, and with/without owner presence. Significant differences in HRV parameters were found between the times of euthanasia. The location of euthanasia, presence of owner, or type of primary diseases had no influence on stress parameters. Horse with colic however presented with less smooth euthanasia. In conclusion, HRV is a sensitive, noninvasive parameter to assess the stress response during euthanasia. Further, euthanasia in horses with colic was the most challenging and modification of the protocols for colic horses should be considered.

**Abstract:**

The effects of a standard protocol for euthanasia on heart rate variability (HRV) as a consequence of stress response were analyzed in this prospective clinical study. The HRV was determined in 40 horses undergoing euthanasia due to various reasons, at different locations, and with/without owner presence. For euthanasia, horses were sedated with xylazine or a combination of xylazine and butorphanol. General anesthesia was induced using diazepam and ketamine. Afterwards, horses were euthanized with pentobarbital. The ECG data were taken by a Telemetric ECG at three time points (sedation, anesthesia, anesthesia until death). The HRV was analyzed including the low (LF) and high frequency (HF) components of HRV and the sympathovagal balance (LF/HF ratio). Significant differences in the LF, HF and LF/HF ratio were found between the three time points of euthanasia (*p* < 0.001). The HRV analysis showed dominating sympathetic activity in the preparation phase of euthanasia and during the injection of pentobarbital. The location of euthanasia, presence of owner and type of primary diseases had no influence on stress parameters. Horses showing excitations or groaning during euthanasia did not differ in HRV. Horse with colic were however more likely to show reoccurrence of breathing during euthanasia. In conclusion, HRV is a sensitive, noninvasive parameter to obtain sympathovagal stimulations during euthanasia and adapted protocols for euthanasia in horse with colic should be studied.

## 1. Introduction

The term “euthanasia” originates from Greek and means “good death.” The process of dying should be as pain- and stress-free as possible for an animal. Consciousness, heart and breathing arrest, and loss of brain function are to be achieved quickly [1]. This “good and smooth” process of dying requires some conditions, according to Auer and Mosing [2]: Reduction and avoidance of excitement, fear and stress before a quickly reached unconsciousness, heart and breathing arrest in deep general anesthesia, assurance of irreversible death and avoidance of danger for all persons involved in the procedure. The idea of this study was based on our clinical impression that euthanasia is stressful for the horse in the majority of cases [3]. Stress factors can be classified in endogenous (i.e., pain associated with colic or fractures, hemodynamic imbalances leading to a shock response) and exogenous factors (i.e., transport to the clinic, foreign people or environment). They lead to an activation of the hypothalamic–pituitary–adrenal-axis, cortisol secretion and sympathetic activation [4]. 

In equine medicine, the analysis of heart-rate variability (HRV) is a noninvasive tool to evaluate the sympathetic–parasympathetic interaction [5]. It has been used in animals exposed to various stimuli to evaluate the effects of medications, response to transport, pain and training [6,7,8,9,10,11], and in horses suffering from cardiac arrhythmias [8,12,13]. The HRV measurement relies on the mathematical analysis of accurately measured variations in successive inter-beat intervals, measured as RR intervals. The high frequency (HF) power of the power spectrum of HRV is thought to reflect primarily parasympathetic nervous activity. Both the sympathetic and the parasympathetic nervous system have been shown to contribute to the low frequency (LF) power component of HRV. Therefore, the LF/HF ratio has been utilized as an index of cardiac sympathovagal balance [14]. 

The aim of this study was to investigate changes in HRV in horses undergoing euthanasia to study their stress level during the procedure. We hypothesized that horses in a more severe hemodynamic status (i.e., horses euthanized for colic) would have a higher activation of the sympathetic nervous system and thus would show a less pronounced changes of HRV after application of drugs used in the standard protocol for euthanasia than horses euthanized for orthopedic reasons. Further, we hypothesized that the presence of the owners or the euthanasia in the stall environment would reduce the stress response in horses under euthanasia.

## 2. Materials and Methods 

### 2.1. Study Population

In this study, HRV analysis was performed in 40 horses of different ages (6–24 years), gender (14 mares, 2 stallions, 24 geldings), weights (380–660 kg) and breeds, during euthanasia, at three times points (U1–U3). The first analysis (U1) took place during sedation, the second (U2) during induction of general anesthesia and the third (U3) during the injection of pentobarbital (U3) until clinical death. Exogenous factors, which might have had an impact on the stress level of the horses, including the type of disease, the indication for euthanasia, the location of the procedure, the presence or absence of the owner and drugs applied, were recorded. A second dosing of pentobarbital was also noted. The procedure of euthanasia (lying down, breathing arrest, auscultation of heart arrest, absence of lid and corneal reflexes) and the behavior (excitations, paddling movements, vocal noises, muscle twitching and recurrence of breathing) were documented in detail. 

### 2.2. Presence of the Owner

In 20 cases, the owner was present during the whole procedure of euthanasia. In the other 20 cases, the owner left before the horse was put down or had agreed to euthanasia on the phone.

### 2.3. Location of Euthanasia

A total of 22 horses were euthanized in a padded stall for anesthesia induction and recovery, which had a swinging door that allowed the fixation of horses against the wall, so that laying down was safe and smooth for the horse and persons involved. Fourteen horses were euthanized in the normal stall of the clinic, as their orthopedic function or general condition did not allow them to be hand walked into the recovery stall. Four horses were euthanized during the first examination in the treatment room. These 18 horses are summarized as “different location” in the following sections. 

### 2.4. Disease Indicating Euthanasia 

Sixteen horses were euthanized due to colic, 12 due to orthopedic problems and 10 due to summarized other diseases, including neoplasia or infections. A total of 19 horses suffered from acute and 21 from chronic disease. 

### 2.5. Medication 

Sixteen horses were sedated with xylazine (0.8 mg/kg body weight (BDW IV), 18 with a combination of xylazine (0.8 mg/kg BDW IV) and butorphanol (0.025 mg/kg BDW IV) and 6 with a combination of detomidine (0.025 mg/kg BDW IV) and butorphanol (0.025 mg/kg BDW IV). General anesthesia using diazepam (0.02 mg/kg i.v.) and ketamine (2.2 mg/ kg BDW IV) was induced in all horses. Afterwards, horses were euthanized with pentobarbital (80 mg/kg BDW IV). 

### 2.6. Heart Rate Variability Analysis

**Figure 1:**
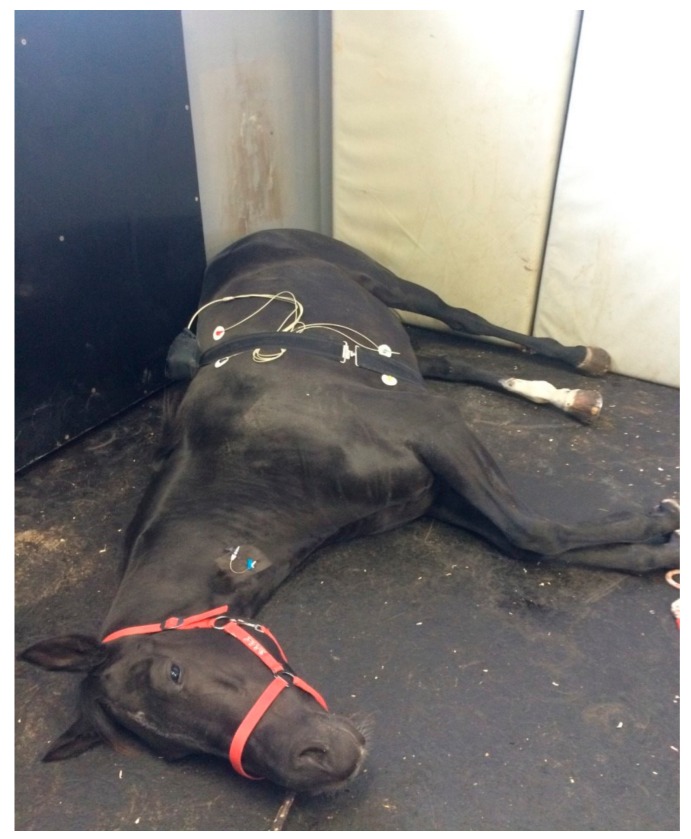
The photograph shows a horse euthanized during the study. The Televet™ ECG is placed within the bag of the abdominal belt.

The original ECG signal was assessed by a telemetric ECG (Televet 100, Fa.Kruuse, Marslev, Denmark). Two adhesive electrodes were placed on the lateral chest wall and one on the sternum, and bipolar chest wall readings were obtained. Sufficient fixation was achieved by an elastic stable girth, which the horses also wore in lateral recumbency during euthanasia (Figure 1). The device was used in the telemetric mode and the data were transmitted via Bluetooth to a notebook and recorded. The ECGs were analyzed by the “Televet™ 100—Software Version 4.1.3” (Company Rösch and Associates Information Engineering GmbH, Frankfurt, Germany) and irregular heartbeats were marked. Medications and abnormal behavior were assigned on the recordings and a comment was filled in. The ECG sequences of 1–5-min length, free of aberrant ECG complexes and artifacts, were cut in three different stages: sedation (U1), general anesthesia (U2) and final injection until death (U3). Regarding HRV analysis, beat-to-beat intervals were imported into the “Heart rate variability Analysis Software 1.1” (Biomedical Signal Analysis Group, University of Kuopio, Finland) and a time and frequency domain analysis was calculated. The HRV, an indicator of the activity of the autonomous nervous system, was studied as described [12,13,14]. The HRV was analyzed in the time domain and expressed as mean heart rate and mean beat-to-beat interval. In addition, the HRV was analyzed in the frequency domain, performing a power spectral analysis by using the Fast Fourier Transformation and calculated as the activity in the HF and LF ranges and as an LF/HF ratio. Frequency component thresholds were set at 0.01–0.07 Hz for LF and 0.07–0.60 Hz for HF. The LF and HF were calculated in normalized units, which allows the comparison of different measurements and subjects. 

### 2.7. Time intervals 

While the first interval, from sedation until the induction of anesthesia, lasted at least 5 min in all horses, there was large variation in the second interval from the induction of anesthesia until euthanasia. The range was from 1–4 min. The third interval began with the injection of pentobarbital and ended with clinical death of the horse. This interval also varied depending on the type of disease, mentality and circulation and the mean duration was about 1 min. 

### 2.8. Statistics

Data were statistically analyzed and graphically presented using SPSS Statistics® (Version 22) and Microsoft® Excel 2010, respectively. The data were tested for normal distribution using the Kolmogorow–Smirnov test and visual inspection. Statistical tests were chosen according to the normality of the data. The level of significance was set at *p* ≤ 0.05. Median and interquartile ranges were calculated for all parameters. 

Comparisons between two time points were carried out using the t-test for independent samples (normal distribution) or Mann-Whitney U test (no normal distribution) with the difference between the time points as a dependent variable and one influence factor as an independent variable. Analysis of variance with repeated measurements (ANOVA) was performed to evaluate the influence of several parameters (phase of euthanasia, euthanasia with owner, kind of disease) on the HRV. The different time points were defined as the repeated factor and either the LF, HF or LF/HF ratio were the dependent variable. A maximum of four influence factors were included in one model due to the sample size. Thus, several models were established. Since sphericity of the models was not given, Greenhouse–Geisser correction was used to estimate within-subject effects. Model diagnostics included visual inspection of the normality and homoscedasticity of the residuals. Although many variables showed no normal distribution, the residuals of the models presented turned out to be sufficient and normal. Two-way ANOVA according to Friedman was used for the evaluation of HRV at the three time points during the procedure. 

## 3. Results

### 3.1. Horses 

Neither age, breed, weight nor sex had a significance influence on the HRV. 

### 3.2. HRV during Euthanasia 

Highly significant differences (*p* < 0.0001, Friedman tests) in HF, LF and LF/HF ratio were found between time periods (Table 1). The first time period was characterized by high LF values (n = 40; median 66.6, interquartile range (IQR) 35.9–118.2 n.u.). Overall, 15 out of 40 horses had an LF/HF ratio above 1.5 n. and, therefore, increased sympathetic activity (n = 40; mean 2.49 ± 4.18 n.u.). In the second phase of euthanasia, a nonsignificant decrease (*p* = 0.172) of the LF/HF ratio in anesthesia was found compared to the first phase. This time period was characterized by significantly increased HF values (*p* = 0.005) and parasympathetic activity dominated (Table 1). The LF also decreased non-significantly from median 66.6 to 36.3 (*p* = 0.172). In the third phase of euthanasia, from the injection of pentobarbital until clinical death, the LF/HF ratio increased significantly compared to the other time periods (*p* < 0.001). The LF activity (median 232.9, IQR 84.8–531.6 n.u.) dominated over HF activity (n = 40; median 59.4, IQR 36.2–84.2 n.u., *p* = 0.001). In this third phase, sympathetic activity dominated (Table 1). Significant differences were found for the LF between the third phase and all other phases (*p* < 0.001, each) and for the HF between the third and the second phase (*p* < 0.001). 

### 3.3. Possible Influencing Stress Factors 

The presence of the owner, the location of euthanasia and the type of disease/indication for euthanasia had no significant influence on the parameters of the HRV (Table 2). The respective multivariable ANOVA analyses with repeated measurement for LF, HF and LF/HF ratio showed that the values between the time periods differed significantly (*p* = 0.001, *p* < 0.001, *p* = 0.046, respectively), but there were no significant effects of the presence of the owner, the location of the euthanasia, the reason for euthanasia or interactions with the time period. 

Horses with colic as the reason for euthanasia (n = 16) generally showed no significant differences to horses with other reasons. The effects were *p* = 0.180 for LF, *p* = 0.961 for HF and *p* = 0.283 for the LF/HF ratio (Table 3).

### 3.4. Sedation before Euthanasia 

Investigation of the sedation revealed generally significant differences between horses sedated with a combination of detomidine and butorphanole (n = 6), with a combination of xylazine and butorphanole (n = 18) and with xylazine alone (n = 16) for LF (*p* = 0.036), but not for HF (*p* = 0.691) or for LF/HF ratio (*p* = 0.781). 

### 3.5. Peculiarities during Euthanasia 

Fifteen horses euthanized due to colic had an increased LF/HF ratio compared to the other horses (Table 3). Four horses, also euthanized due to colic, showed a reoccurrence of breathing after respiratory arrest (Table 4). The decrease of LF and LF/HF ratio from sedation to induction of anesthesia were significantly stronger in these horses compared to the others (*p* = 0.016 in both tests, Mann-Whitney U test). A respective significant increase could be shown for HF (*p* = 0.030, t-test). There were, however, no significant differences in horses that showed excitations and paddling movements during euthanasia (n = 4, *p* = 0.820, *p* = 0.444, *p* = 0.682, univariable ANOVA with repeated measurements LF, HF and LF/HF ratio, respectively), groaning or other noises (n = 16, *p* = 0.167, *p* = 0.236, *p* = 0.595, univariable ANOVA with repeated measurements for LF, HF and LF/HF ratio, respectively), diffuse cutaneous fasciculations (n = 11, *p* = 0.529, *p* = 0.831, *p* = 0.672, univariable ANOVA with repeated measurements for LF, HF and LF/HF ratio, respectively) or final gasps (n = 15, *p* = 0.572, *p* = 0.938, *p* = 0.215, univariable ANOVA with repeated measurements for LF, HF and LF/HF ratio, respectively) (Table 5). 

## 4. Discussion

### 4.1. Data of HRV during Euthanasia 

In the study presented, LF was almost similar to HF in the first phase of euthanasia in all horses despite sedation using xylazine and detomidine, indicating a predominant sympathetic activity. A possible explanation, apart from pain and excitement, might be the shortness of the time interval in which ECG data was collected, as alpha-2-agonists stimulate peripheral alpha-receptors first leading to blood pressure increase for 1–2 min. After this, centrally mediated and long lasting decrease in blood pressure and a bradycardia occurs [2]. The first time interval, in which EGC data was collected, lasted 5 min in all horses. Therefore, the increased sympathetic activity in this time interval might be the consequence of the alpha-2-agonist-induced initial hypertension (due to peripheral postsynaptic adrenoreceptors causing vasoconstriction), which results in a baroreceptor-mediated reflex bradycardia. The veterinarian performing the euthanasia decided on induction of anesthesia and, therefore, the beginning of the second phase of euthanasia. Clinical signs, such as hanging head, neck and ears, led to injection of anesthetics. Assurance of a reduction in heart rate was not commonly performed. In future studies to evaluate stress in the preparation of euthanasia, it might be useful to separate this preparation phase into placement of the catheter and walking the horse to the location of euthanasia and the phase in which the horse is sedated before the induction of anesthesia. In the second time interval, horses were in general anesthesia. Parasympathetic activity clearly dominated in this phase. The reason for this might the analgesia as part of the general anesthesia when the body reaches a relaxed state. Apart from this, the drugs applied have an influence on the heart rate and the HRV. Ketamine leads to a secretion of catecholamines and increased heart rate [15]. This phenomenon might be suppressed by the alpha-2-agonists applied earlier, as these decrease the central sympathetic tone, the blood pressure and the heart rate [2]. The diazepam, injected in combination with ketamine maybe cause a depression of the limbic system [16]. This “anxiolytic effect” decreases excitement, fear and stress in the organism and its psychosedative effect leads to a reduction in the heart rate and an increase of the HRV [16].

The last phase of euthanasia, from injection of pentobarbital until clinical death, was characterized by a significant increase of sympathetic activity and, therefore, reduced HRV. This is the consequence of the tachycardia induced by pentobarbital. The cardiac preload decreases due to the vasodilatory effect of pentobarbital, while a compensatory increase of the heart rate is noted due to the baroreceptor reflex [15]. Death occurs as a consequence of overdosing, leading to respiratory and cardiac arrest [1].

### 4.2. Peculiarities during Euthanasia 

There were no significant differences in the HRV in horses which showed excitations (n = 4), groaning (n = 16) and diffuse cutaneous fasciculations (n = 11). Nevertheless, horses euthanized due to acute disease showed significantly more cutaneous fasciculations (*p* = 0.04) than those euthanized due to chronic problems. Four horses, also euthanized due to colic, showed agonal breaths after respiratory arrest. The LF and LF/HF ratio from sedation to induction of anesthesia were significantly increased in these horses (*p* = 0.03, *p* = 0.04, respectively). This might indicate increased stress in these four horses. Apart from the vagosuppressive effect of the drugs applied, this stress might be caused by visceral pain and hemodynamic imbalances, leading to increasing heart rate and the LF component. As only colic patients showed this phenomenon, it might be assumed that colic can increase the incidence of reoccurrence of breathing compared to other horses. This hypothesis should be substantiated by further studies in a larger number of horses, so that the owners could be prepared for this scenario in horses suffering from severe colic leading to euthanasia. Another negative scenario for the owner is the occurrence of final gasps during euthanasia. This terminal breathing is caused by the discrepancy in the sensitivity of the breathing center in the medulla oblongata and the sensitivity of the cortex concerning oxygen supply [17]. Fourteen horses showed final gasps in the study presented. No significant differences in the HRV were found in comparison to other horses. Nevertheless, this phenomenon is stressful and hard to understand for the owner present. Therefore, a finding in dogs, where Evans et al. [17] found that fewer final gasps occurred with the use of lidocaine (4.4 mg/kg BDW) in combination with pentobarbital, should be studied in horses, especially those euthanized due to colic, presenting a concurrence of severe pain and hemodynamic imbalance. 

### 4.3. Effect of Drugs 

Each of the 40 horses included into this study was under the influence of different drugs, depending of the type of disease prior to euthanasia. Eleven horses had been treated by the referring veterinarian before the transport to the clinic, where they were finally euthanized. It can only be speculated if and to which extent these drugs may have influenced data obtained in the presented study, as research is rare on the effect of various drugs on the HRV [18]. The group of colic patients (n = 16) had all been treated with metamizole and/or flunixin-meglumine by the referring veterinarian or in the clinic to achieve analgesia in combination with spasmolytic butylscopolamin. As the general condition was very bad in all colic patients prior to euthanasia, the alpha-2-agonist xylazine and/or the opioid butorphanol were also commonly applied. Drugs that reduce sympathetic effects, pain and inflammatory reactions have a positive influence on the HRV. The influence of metamizole and flunixin-meglumine leads to an increase of the HF and decrease of the LF component with the consequence of increasing the HRV. The commonly postoperatively used acetylcholinesterase blocker and motility increasing constigmine also have this positive influence on the HRV [19]. For the record, nonsteroidal anti-inflammatory drugs have a proven positive effect on the HRV, while the influence of other drug groups, for example, antibiotics, remains to be studied. As the horses included in this study had received various drugs due to their primary disease, it was impossible to evaluate the drugs’ effects on the HRV in detail. A further limitation is that we had no baseline HRV measurements form the horses included into the study. However, we were more interested in the changes in HRV in each animal over the 3 measurements time points rather than in absolute values. Therefore, we believe that the present results can be used in assessing difference in underlying causes for euthanasia, the presence or absence of the owners well as the effect of different locations. Nevertheless, intraindividual differences were found during euthanasia, which substantiates the feasibility of the HRV as a stress response indicator despite the use of various drugs. The effects of drug type, dosage, timing and application route should be evaluated in further studies. Similarly, it should be taken into consideration whether an adapted protocol for euthanasia in horses with colic is needed 

## Figures and Tables

**Table 1:** Median and Interquartile Range (IQR) of low frequency (LF), high frequency (HF) and LF/HF ratio in all 40 euthanized horses in the first (sedation), second (anesthesia) and third (anesthesia until respiratory arrest) phase of euthanasia.

Phase of Euthanasia	LF (n.u.)	HF (n.u.)	LF/HF Ratio (n.u.)
1st phase (median (IQR))	66.6 (35.9–118.2) ^a^	71.4 (50.5–88.7) ^a^	0.97 (0.40–2.42) ^a^
2nd phase (median (IQR))	36.3 (10.8–54.5) ^b^	91.9 (81.6–94.8) ^a,b^	0.46 (0.11–0.63) ^b^
3rd phase (median (IQR))	232.9 (84.8–531.6) ^a,b^	59.4 (36.2–84.2) ^b^	2.8 (1.4–13.5) ^a,b^

n.u.= normal units; ^a,b^ —columns with the same superscript letters showed significant differences.

**Table 2:** Median and IQR of LF, HF and LF/HF ratio in all 40 euthanized horses in the first (sedation), second (anesthesia) and third (anesthesia until respiratory arrest) phase of euthanasia with and without the owner present.

Phase of Euthanasia	LF (n.u.)		HF (n.u.)		LF/HF Ratio (n.u.)	
	With Owner	Without Owner	With Owner	Without Owner	With Owner	Without Owner
1st phase (median (IQR))	66.95 (36.55–127.15)	66.60 (37.40–113.10)	72.7 (53.50–88.25)	70.25(47.00–89.05)	1.021 (0.423–2.512)	0.9735 (0.4150–2.4085)
2nd phase (median (IQR))	34.00 (7.80–47.10)	36.35 (12.65–58.95)	90.05 (82.10–96.00)	92.75(81.10–94.70)	0.395 (0.0805–0.585)	0.427 (0.135–0.636)
3rd phase (median (IQR))	227.50 (74.80–486.10)	233.65(104.35–588.95)	62.05 (32.45–85.20)	58.75 (43.80–79.45)	5.756 (1.1225–16.0415)	5.5165 (1.455–12.839)

LF = low frequency; HF = high frequency; n.u.= normal units

**Table 3:** Median and IQR of LF, HF and LF/HF ratio in all 40 euthanized horses in the first (sedation), second (anesthesia) and third (anesthesia until respiratory arrest) phase of euthanasia comparing colic versus orthopedic disease as the reason for euthanasia.

Phase of Euthanasia	LF (n.u.)		HF (n.u.)		LF/HF Ratio (n.u.)	
	Colic (n = 16)	Orthopedic Disease (n= 12)	Colic (n = 16)	Orthopedic Disease (n = 12)	Colic (n = 16)	Orthopedic Disease (n= 12)
1st phase (median (IQR))	66.6 (37.2–153.6)	68.5 (28.1–111.1)	68.4 (49.6–85.8)	77.1 (53.5–89.0)	1.14 (0.50–2.58)	0.96 (0.33–2.09)
2nd phase (median (IQR))	36.4 (7.7–57.6)	31.4 (25.4–54.6)	88.7 (77.7–94.2)	87.7 (74.7–93.6)	0.47 (0.08–0.64)	0.35 (0.28–0.64)
3rd phase (median (IQR))	324.7 (75.2–755.7)	228.0 (111.0–475.7)	60.3 (39.2–86.8)	59.7 (43.8–75.7)	8.94 (0.88–13.42)	5.52 (1.51–13.83)

LF = low frequency; HF = high frequency; n.u.= normal units

**Table 4:** Median and IQR of LF, HF and LF/HF ratio in horses in the first (sedation), second (anesthesia) and third (anesthesia until respiratory arrest) phase of euthanasia with peculiarities during euthanasia (n = 12), reoccurrence of breathing (n = 4) and second dosing of pentobarbital (n = 2).

1st Phase of Euthanasia	LF (n.u.)	HF (n.u.)	LF/HF Ratio (n.u.)
Peculiarities during euthanasia (n = 12)	61.9 (42.8–88.6)	65.3 (57.8–83.4)	0.97 (0.55–1.59)
Reoccurrence of breathing (n = 4)	236.6 (93.1–426.1)	47 (33.0–66.2)	6.17 (1.66–13.78)
Second dosing of pentobarbital (n = 2)	297.1 (107.5–486.6)	47.0 (45.1–48.9)	6.17 (2.38–9.95)
**2nd Phase of Euthanasia**			
Peculiarities during euthanasia (n = 12)	51.3 (39.6–63.7)	84.1 (77.8–93.4)	0.62 (0.44–0.71)
Reoccurrence of breathing (n = 4)	29.3 (17.3–37.3)	92.2 (88.8–95.9)	0.33 (0.20–0.39)
Second dosing of pentobarbital (n = 2)	17.3 (7.7–26.9)	90.4 (87.0–93.8)	0.20 (0.08–0.31)
**3rd Phase of Euthanasia**			
Peculiarities during euthanasia (n = 12)	157.6 (79.9–787.9)	59.8 (44.1–83.3)	3.38 (1.24–13.42)
Reoccurrence of breathing (n = 4)	376.2 (267.8–589.0)	31.4 (18.1–51.9)	12.08 (9.32–18.58)
Second dosing of pentobarbital (n = 2)	480.6 (243.3–717.9)	38.6 (17.8–59.3)	12.08 (12.05–12.11)

LF = low frequency; HF = high frequency; n.u.= normal units.

**Table 5:** Mean values and standard deviation (SD) of LF, HF and LF/HF ratio in horses in the first (sedation), second (anesthesia) and third (anesthesia until respiratory arrest) phase of euthanasia showing groaning (n = 16), excitations (n = 4) and diffuse cutaneous fasciculations (n = 11) during euthanasia.

1st Phase of Euthanasia	LF (n.u.)	HF (n.u.)	LF/HF Ratio (n.u.)
Groaning (n = 16)	83.3 (41.7–128.2)	65.2 (44.2–87.7)	1.50 (0.48–2.72)
Excitations (n = 4)	83.3 (40.9–172.1)	58.2 (39.6–85.0)	1.50 (0.65–4.42)
Diffuse cutaneous fasciculations (n = 11)	62.2 (31.4–116.6)	72.3 (48.9––86.6)	0.86 (0.37–2.38)
**2nd Phase of Euthanasia**			
Groaning (n = 16)	19.8 (8.5–37.6)	92.8 (79.9–94.6)	0.21 (0.09––0.48)
Excitations (n = 4)	21.2 (11.2–61.0)	93.4 (72.7–95.2)	0.23 (0.12––1.03)
Diffuse cutaneous fasciculations (n = 11)	26.9 (7.7–59.7)	93.8 (87.0–96.5)	0.31 (0.08–0.66)
**3rd Phase of Euthanasia**			
Groaning (n = 16)	228.3 (53.4–411.4)	51.7 (17.6–80.8)	6.21 (0.60–13.84)
Excitations (n = 4)	238.7 (171.7–491.7)	45.3 (24.9–74.65)	9.47 (4.00–13.76)
Diffuse cutaneous fasciculations (n = 11)	445.3 (130.2–717.9)	59.3 (32.4–84.8)	12.11 (1.43–23.23)

LF = low frequency; HF = high frequency; n.u.= normal units.
